# Nudging Online Gamblers to Withdraw Money: The Impact of Personalized Messages on Money Withdrawal Among a Sample of Real-World Online Casino Players

**DOI:** 10.1007/s10899-023-10276-1

**Published:** 2023-12-18

**Authors:** Michael Auer, Mark D. Griffiths

**Affiliations:** 1 Neccton GmbH, Gertrude-Fröhlich-Sandner-Strasse, 21100 Vienna, Austria; 2 International Gaming Research Unit, Psychology Department, Nottingham Trent University, 50 Shakespeare Street, Nottingham, NG1 4FQ UK

**Keywords:** Gambling, Responsible gambling, Nudging, Withdrawing money, Personalized messaging

## Abstract

A number of scholars have argued that online gambling can be more problematic than land-based gambling. Motivating gamblers to withdraw money from their online gambling account could lower losses because there would be less money available to lose. Therefore, the present study investigated whether personalized messages are an effective way of ‘nudging’ gamblers to withdraw money from their online gambling account. The authors were given access to a secondary dataset by *Nederlandse Loterij* (the national Dutch Lottery operator) comprising 4049 online gamblers. Two types of messages were used to ‘nudge’ gamblers to withdraw money from their gambling account (i.e., a ‘winning streak’ message and a ‘withdrawal’ message). The findings indicated that (i) 38% of gamblers reading the ‘winning streak’ messages withdrew money from their gambling account on the same day, and (ii) 18% of gamblers reading the ‘withdrawal’ messages withdrew money from their gambling account on the same day. Gamblers who read personalized messages also withdrew larger amounts of money from their gambling accounts compared to gamblers who did not read personalized messages. The findings suggest that the personalized messages can have an impact on both the likelihood to withdraw money as well as the amount of money which was withdrawn and could help reduce gambling-related harm.

## Introduction

Over the past two decades, online gambling and problematic online gambling have significantly increased (Lawn et al., 2020). Many studies have asserted that online gambling is associated with a higher likelihood of problem gambling compared to land-based gambling (e.g., Effertz et al., 2018; Griffiths et al., 2006; McBride & Derevensky, 2009; Petry, 2006; Yazdi & Katzian, 2017). More specifically, studies have reported that the prevalence of online problem gambling is three to eight times higher than the prevalence of land-based gambling (Chóliz et al., 2019; Effertz et al., 2018; Griffiths et al., 2009; Volberg et al., 2018). However, most online gamblers also gamble offline (Wardle et al., 2011). Therefore, it has been argued that it is not the online medium itself that is problematic but that for vulnerable individuals (e.g., those with gambling problems), the internet can be inherently more ‘dangerous’ because of factors such as its 24/7 accessibility (Wardle et al., 2011).

According to Griffiths (2003), a number of situational and structural factors can make online gambling potentially more problematic (e.g., accessibility, event frequency, convenience, anonymity). Among the more important factors is accessibility. Online gambling is more accessible than land-based gambling because gamblers no longer need to travel to a location due to the advent of Wi-Fi-enabled devices such as smartphones, tablets, laptops, and computers. Anonymity is another important aspect in the maintenance of online gambling because it allows individuals to privately engage in gambling without the fear of stigma.

### Accessibility, Event Frequency, and Problem Gambling

Several studies have reported that the accessibility of gambling is associated with an increased prevalence of problem gambling (e.g., González-Roz et al., 2017; St-Pierre et al., 2014). In a cross-sectional study, Barratt et al. (2014) found that the availability of land-based gambling machines correlated with the individuals seeking help for their gambling problems. In a survey of 2,631 US adults, Welte et al. (2004) found that the presence of a casino within 10 miles of the participant’s home was positively associated with problem gambling. Other studies have found that specific forms of gambling, mainly electronic game machines (EGMs), have the potential to be more addictive than other types of gambling (Auer & Griffiths, 2023; Brooks et al., 2008; Dowling et al., 2005; Griffiths, 1993, 2008; Livingstone & Woolley, 2008). Outside of an individual’s vulnerabilities, this is mostly a consequence of the high event frequency of EGMs. In online gambling, individuals can wager on multiple games simultaneously which further increases event frequency (Brosowski et al., 2012; Griffiths et al., 2010).

Moreover, not only do some researchers argue that online gambling in itself is not problematic, but that other factors are involved such as individuals’ vulnerabilities and susceptibilities (e.g., being an adolescent, being a problem gambler, having comorbid disorders) (Griffiths & Calado, 2022). Studies have found that those who have already problems with gambling are more likely to gamble online (Emond et al., 2020; Wijesingha et al., 2017; Yazdi & Katzian, 2017). This may also explain the higher prevalence of problem gambling among online gamblers (i.e., these are gamblers that will gamble in any medium and are not online gamblers *per se*).

### Prevalence of Problem Online Gambling

Chóliz (2016) examined the effect of online gambling in Spain two years after its legalization. The sample included 1277 pathological gamblers in recovery at 26 gambling addiction treatment centers. The study reported a significant increase in young pathological gamblers since the legalization of online gambling in Spain. Chóliz et al. (2021) were given access to data from 6816 gamblers by the Spanish General Directorate of Gambling Regulation. They found that young adults (those under the age of 35 years) participated significantly more in online gambling than gamblers aged between 35 and 65 years and elderly people (> 65 years). Furthermore, the prevalence of pathological gambling among gamblers who had also gambled online was 7.26%, whereas among those who had not gambled online it was 0.69%. The percentage of problem gamblers among online gamblers was therefore 10 times higher than for those who did not gamble online. This was identical to an earlier study by Griffiths et al. (2009) who reported that among a representative sample of the British population, the prevalence of problem gambling was 5% among those who had gambled online but only 0.5% who had only ever gambled offline.

Recently, Mora-Salgueiro et al. (2021) conducted a systematic review of online problem gambling. Their review initially resulted in 427 studies but only 20 of these were evaluated based on pre-determined criteria. However, the 20 studies assessed problem gambling differently and also defined online gambling differently. Despite these problems, the authors still identified a few important findings. The studies reported a prevalence of disordered online gambling between 2.7% and 20.3%, and among adolescents the prevalence for at-risk and problem gambling was between 5.7% and 57.52%. Being single and being male were the most common risk factors for online problem gambling.

### Online Gambling and Responsible Gambling Tools

Although there is a growing literature concerning online problem gambling, online gambling facilitates opportunities for safter gambling that are unavailable offline in the form of responsible gaming tools. Online gambling operators know every transaction of their clientele from the moment the gambler first registered. This includes their bets, wins, deposits, and withdrawals. Consequently, operators have a complete picture of a gambler’s behavior. Furthermore, they can potentially interact with gamblers at any time via pop-up messages, personalized messages, text messages, e-mails and/or telephone calls (Auer & Griffiths, 2015).

A number of studies have investigated problem gambling risk mitigating tools in online gambling. Several studies have shown that voluntary or mandatory limit setting can reduce subsequent losses (e.g., Heirene & Gainsbury, 2021; Ivanova et al., 2019). Voluntary self-exclusion is another popular responsible gaming tool that most gambling operators now provide to their players. Based on a sample of gamblers from a Swedish treatment facility, Håkansson and Akesson (2022) found that many patients with gambling problems had voluntarily self-excluded. Håkansson and Akesson (2022) concluded that voluntary self-exclusion was a commonly used by problem gamblers.

Auer and Griffiths (2023) argued that gamblers need bespoke feedback regarding their financial expenditure because they frequently underestimate the amount of money they deposit when gambling. Consequently, a growing number of studies have investigated the impact of personalized feedback on subsequent gambling behavior (e.g., Auer & Griffiths, 2015, 2020; Focal Research, 2004; Schellink & Schrans, 2002). Such personalized feedback can be provided to players numerically, graphically, verbally, and in written form (via text messages, emails or in-game pop-ups). Several studies have found that personalized inbox messages on online casino websites led to a subsequent reduction in money spent (e.g., Auer & Griffiths, 2015, 2020). It is assumed that this behavioral change can be a consequence of such psychological processes as cognitive dissonance which can be triggered by feedback (Auer & Griffiths, 2020). Other studies found that feedback information on video lottery terminals (VLTs) via on-screen pop-ups can lead to decreased monetary spending (Focal Research, 2004; Schellink & Schrans, 2002).

A number of studies have investigated the impact of warning messages and personalized messages on EGMs in the early 2000s (e.g., Benhsain et al., 2004; Focal Research, 2004; Ladouceur & Sevigny, 2003; Schellink & Schrans, 2002). In an analysis of responsible gambling features on VLTs in Canada, Schellink and Schrans (2002) concluded that to help gamblers keep in control, on-screen clocks and expenditure balances should permanently be permanently displayed. They also found that when asked, 90% of players remembered the appearance of responsible gambling pop-up messages on VLTs while gambling. Monaghan (2008) reviewed the existing literature at that time and concluded that there was support for the introduction of pop-up messages including breaks in play as a responsible gambling strategy. Monaghan argued that these strategies may modify gambling-related cognitions and behavior.

As aforementioned, studies have reported that gamblers (particularly those that gamble very regularly) underestimate their financial losses (Auer & Griffiths, 2017; Heirene et al., 2022). Four studies have compared self-reported spending with actual spending among real-world samples of online gamblers (Auer & Griffiths, 2017; Braverman et al., 2014; Heirene et al., 2022; Wohl et al., 2017). All four studies concluded that gamblers under-estimated their losses and/or overestimated their winnings. For example, Heirene et al. (2022) reported that 64.9% of online sports bettors underestimated their losses in a 30-day period. Braverman et al., (2014) reported that between 34% and 40% of gamblers underestimated their losses and overestimated their winnings. Auer and Griffiths (2017) identified a discrepancy between self-reported losses and actual losses in relation to gambling intensity. The higher the amount wagered during the past 30 days the more gamblers underestimated their actual losses. Wohl et al. (2017) asked land-based gamblers how much they had won or lost over a three-month period using data from their loyalty cards. Results indicated that gamblers who under-estimated their losses significantly reduced the amount they wagered as well as the amount they lost during the three-month follow-up period.

Studies have also indicated that feedback regarding monetary spending might increase gamblers’ awareness of their own behavior. Auer and Griffiths (2015) analyzed the impact of personalized feedback regarding money lost and time spent, as well as the impact of personalized messages on subsequent money wagered. The study was based on 1015 real-world online gamblers which had voluntarily registered to use a player tracking tool. Personalized messages informed gamblers about increased money or time spent. Based on objective player tracking data, they found that gamblers who viewed the information subsequently spent less money gambling. Similarly, in a study of 7314 Swedish online gamblers who read feedback concerning their own actual gambling behavior in the form of text messages also found that the amount wagered significantly decreased seven days after receiving the message (Auer & Griffiths, 2020). In that study, the messages addressed various types of behavior including high losses, long play duration, increased deposits, and increased playing frequency. One personalized message was sent to gamblers who had recently had a winning streak and recommended that gamblers should withdraw some of their winnings. These messages led to a significant reduction in the amount of money gambled on the day it was read.

### The Present Study

The present study investigated two types of personalized messages (‘nudges’) which are sent to actual online gamblers after they have won a large amount of money. Similar messages were studied by Auer and Griffiths (2020) in a different sample of online gamblers. Nudge theory was proposed by Thaler and Sunstein (2009) and posits that outside forces can subtly guide an individual’s decision in one direction or another. More specifically:

*A nudge…is any aspect of the choice architecture that alters people’s behavior in a predictable way without forbidding any options or significantly changing their economic incentives. To count as a mere nudge, the intervention must be easy and cheap to avoid. Nudges are not mandates. Putting fruit at eye level counts as a nudge. Banning junk food does not* (Thaler & Sunstein, 2009).

Nudging has been successfully used to decrease unhealthy food consumption (Arno & Thomas, 2016) and energy consumption (Gillingham & Tsvetanov, 2018) but not gambling consumption. Gainsbury et al. (2020) described a framework of using behavioral science to reduce gambling-related harm. They noted that behavioral economics research could be used to identify nudges that might help maintain healthy levels of gambling without restricting the autonomy of players. Newall (2019) claimed that gambling operators used nudging techniques to increase the time individuals spend gambling spent and that this may increase problematic gambling behavior.

The present study investigated whether personalized messages are an effective way of ‘nudging’ gamblers to withdraw money from their online gambling account. Previous real-world studies have investigated whether online casino gamblers spend less money after reading personalized messages which were provided by the online gambling operator (i.e., Auer & Griffiths, 2015, 2020). In order to gamble, money has to be transferred from a bank account, credit card or any other source into the online gambling account. Motivating gamblers to withdraw money from their gambling account could lower losses because there is less money available to lose. The present study tested the following hypothesis: gamblers who read specific personalized messages are more likely to withdraw money from their gambling account.

## Method

### Participants

The authors were given access to a secondary dataset by *Nederlandse Loterij* (the national Dutch Lottery operator) comprising 4049 online gamblers. *Nederlandse Loterij* offers casino games such as slots, roulette, blackjack, and sports betting. Each of the 4049 gamblers read one or two responsible gambling messages between October 1st, 2022 and January 31, 2023. The authors had access to every single bet, win, monetary deposit, and money withdrawal for the seven days before a message was read and on the day a message was read.

### Personalized Messages

*Nederlandse Loterij* uses the player tracking software *mentor* (Auer & Griffiths, 2015, 2020). Gamblers can access information regarding their bets, wins, monetary deposits, and money withdrawals in a dedicated section of the online gambling site. There, they can also retrieve personalized messages which are triggered by specific gambling behavior. Gamblers have to actively navigate to the respective section and click on a message to see the full text. This action is tracked with the respective date and time of day. A gambler can receive (at most) one message per week and the same message is not sent more than once during a three-month period. Two types of messages are used to ‘nudge’ gamblers to withdraw money from their gambling account (i.e., a ‘winning streak’ message and a ‘withdrawal’ message).

‘Winning streak’ message: *Happy to see that you have recently won! Why don’t you use some of that money on a nice dinner or buy yourself something you want? Otherwise, it could be gone faster than you think*.

This message is sent if a gambler wins more than €250 the day before.

‘Withdrawal’ message: *It seems like you rarely cash out, even when you win. Withdrawing some money after a win can help you avoid spending more than you can afford*.

This message is sent if a gambler meets the following four criteria (i.e., all four criteria have to have occurred before a message is sent to the gambler):

- Deposited at least €500 over the past 30 days
- Less than 20% of that amount of money has been withdrawn
- Gambled on at least five days during the past seven days
- Lost money the day before.

A loss means that the amount of money won was lower than the amount of money bet. Gamblers do not have to read messages on the day they are sent and their gambling profile might be different on the day a message is read. For that reason, the number of days between message sent and reading was limited to seven days. The amount of money bet, lost and deposited during the seven days before a message was read was computed. Also, the amount of money withdrawn on the day a message was read was also computed.

### Matched Pairs Design

The goal of the study was to evaluate whether the reading of a personalized message by gamblers led to money withdrawals from their online gambling account. However, it is not valid to simply report the percentage of gamblers which withdrew money before and after personalized messages were read. The authors considered various analytical approaches and in line with previous similar studies (e.g., Auer & Griffiths, 2015, 2020, 2022a) chose a matched-pairs design. In this approach each gambler along with the date of reading a message was matched with a gambler that did not read a message on that day, but was similar with respect to a number of demographic and gambling behavior criteria. The matching procedure was as follows:

- *Age:* The age difference between the gambler reading a message and a matched gambler was at most five years apart.
- *Gender:* A matched gambler had to have the same gender as a gambler reading a message.
- *Amount of money bet in the seven days before a message was read:* A matched gambler had to bet at least 90% and at most 110% compared to the gambler who read a message. If a gambler reading a message bet €1000 in the seven days before, a matched gambler had to have bet between €900 and €1100 in the seven days before.
- *Amount of money deposited in the seven days before a message was read:* A matched gambler had to deposit at less 90% and at most 110% compared to the gambler who read a message. If a gambler reading a message deposited €100 in the seven days before, a matched gambler had to have deposited between €90 and €110 in the seven days before.
- *Amount of money lost in the seven days before a message was read:* The amount of money lost was computed as the difference between the amount of money won and the amount of money bet. A negative value indicates a loss and a positive value indicates a win. A matched gambler had to lose/win at least 90% and at most 110% compared to the gambler who read a message. If a gambler reading a message won €500 in the seven days before, a matched gambler had to have won between €450 and €550 in the seven days before. If a gambler reading a message lost €400 in the seven days before, a matched gambler had to have lost between €360 and €440 in the seven days before.

Every gambler who received a message could be matched with none up to any number of gamblers based on the above listed criteria. If a gambler who received a message was matched with more than one individual, the one gambler with the most similar amount deposited was chosen. This led to at most one matched gambler for each gambler that received a message. Gamblers reading a message could of course also have had no match.

### Data Analysis

Differences between independent observations not following a normal distribution were tested using non-parametric Mann–Whitney U-tests (MacFarland & Yates, 2016). Differences between dependent observations not following a normal distribution were tested using non-parametric Wilcoxon signed-rank tests (Cuzick, 1985). Z-tests were used to compare percentages between different independent groups (Lawley, 1938).

## Results

Out of the 4,049 gamblers of the *Nederlandse Loterij* who read one or more personalized message between October 1st, 2022 and January 31, 2023, (i) 3761 gamblers read one message between October 1st, 2020 and January 31st, 2023, (ii) 263 gamblers read two messages, and (iii) 25 gamblers read three messages. The 4049 gamblers produced 4362 observations (where each observation was a combination of gambler, message type, and date). Out of the 4362 observations 1663 were able to be matched with at least one gambler that did not read a message (38%).

Table 1 reports the average age, percentage of females, median amount of money deposited, and median amount of money bet, as well as the median amount of money lost/won seven days before reading a message for observations which could be matched and observations which could not be matched. Unmatched gamblers were significantly older (T =  − 12,* p* < 0.001) and the percentage of females (Z = − 8, *p* < 0.001) was significantly higher. Mann–Whitney *U*-Tests reported significant differences between matched and unmatched observations with respect to amount deposited (U = 2,696,521, *p* < 0.001), amount bet (U = 1,983,376, *p* < 0.001) and amount lost/won (U = 1,260,651, *p* < 0.001).

**Table 1:** Average and median values for matched and unmatched observations

	N	Age	Female	Median amount of money deposited seven days before	Median amount of money bet seven days before	Median amount of money lost/won seven days before
Unmatched	2699	40.16	16%	€128	€1232	€106.52
Matched	1663	36.19	8%	€200	€894	− €110.74

Unmatched gamblers deposited less money, but bet more. Unmatched gamblers on average won €106 and matched gamblers on average lost €110. However, the loss is a volatile metric. It converges towards the percentage which is paid out based on the amount bet, but for a low number of observations it can display a large volatility. In the present study, the loss was computed for each of the observations based on the seven days before reading a message. Consequently, each computed value was derived from a relatively small number of data points and it can be positive or negative. The fact that the median loss was positive for unmatched gamblers and negative for matched gamblers does not carry significant meaning.

Out of the 1663 observations that were matched, 372 read the ‘winning streak’ message and 1291 read the ‘withdrawal’ message. The messages are based on different computations and the difference between the frequency does not carry any meaning. Gamblers reading the ‘winning streak’ message were significantly younger (*t* =  − 5.27, *p* < 0.001) and more frequently male (Z =  − 4.12, *p* < 0.001). Gamblers reading the ‘winning streak’ message deposited significantly less money (U = 186,541, *p* < 0.001) and bet significantly less money (U = 225,612, *p* < 0.001) in the seven days before. In the seven days before gamblers read the ‘winning streak’ message, they won on average €249. This is in line with the reason for this message because gamblers who won €250 in the previous days received the message. Gamblers who read the ‘withdrawal’ message on average lost €146 in the seven days before. The difference between the two message groups with respective to loss/win was also significant (U = 398,592, *p* < 0.001) (Table 2).

**Table 2:** Average and median values for matched observations by message type

	N	Age	Female	Median amount of money deposited seven days before	Median amount of money bet seven days before	Median amount of money lost/won seven days before
‘Winning streak’ message	372	34.05	3%	€150	€748.42	€249
‘Withdrawal’ message	1291	36.81	10%	€215.78	€921.23	− €146.7

In order to determine whether the reading of messages had an impact on money withdrawal, the matched observations had to be taken into account. Each of the 372 and 1291 observations was matched with one gambler not reading a message on the same day. In 143 cases, gamblers who read the ‘winning streak’ message withdrew money from their gambling account on the same day (38%). Only 52 of the respective matched gamblers withdrew money (14%). The difference was significant (Z = 7.88, *p* < 0.001). The respective numbers for gamblers for the ‘withdrawal’ message were 236 individuals (18%) and 131 individuals (10%). The difference was also significant (Z = 5.96,* p* < 0.001) (Table 3).

**Table 3:** Number of observations with a withdrawal on the day a message was read and respective numbers for the matched observations

	N	Number of observations with withdrawal	Number of matched observations with withdrawal
‘Winning streak’ message	372	143 (38%)	52 (14%)
‘Withdrawal’ message	1291	236 (18%)	131 (10%)

Next, it was tested whether reading a message had an impact on the amount of money withdrawn. The median amount withdrawn was computed based on the observations where a withdrawal actually took place. Gamblers who read the ‘winning streak’ message and actually withdrew money, withdrew on average €400. The matched gamblers who withdrew money, withdrew on average €165. This difference was significant (W = 15,001, *p* < 0.001; Wilcoxon test). Gamblers reading the ‘withdrawal’ message and withdrawing money, on average withdrew €200 and the matched gamblers average withdrawal amount was €100. Again, this difference was significant (W = 2046, *p* < 0.001; Wilcoxon test). For both messages read, the amount of money withdrawn by gamblers reading a message was larger than for the matched observations (Table 4).

**Table 4:** Median withdrawal amount for gamblers which withdrew on the day a message was read and the respective matched observations

	Actual observations		Matched observations	
	N read message	Median amount of money withdrawn	N read message	Median amount of money withdrawn
‘Winning streak’ message	143	400	52	165
‘Withdrawal’ message	236	200	131	100

In order to test whether there was a difference with respect to gambling intensity, the observations where split into four groups based on the amount of money deposited in the previous seven days. Table 5 reports the number and percentage of gamblers who withdrew money for each intensity group as well as the respective number for the matched observations. Table 5 reports the results for the ‘winning streak’ message. For each intensity group, the percentage was larger among gamblers who read the ‘winning streak’ message compared to the matched observations. The smallest difference occurred among the group of most intense gamblers. This difference was not significant. However, the differences among the other three groups were significant.

**Table 5:** Percentage of gamblers withdrawing split into four groups based on the amount of money deposited seven days before. Results for the ‘winning streak’ message

Intensity group	N	Minimum amount of money deposited seven days before	Maximum amount of money deposited seven days before	Number of observations with withdrawal	Number of matched observations with withdrawal	Z	*p*
1	95	10	70	43 (45%)	10 (11%)	5.76	< 0.001*
2	104	75	150	42 (40%)	17 (16%)	3.97	< 0.001*
3	80	160	270	33 (41%)	9 (11%)	4.56	< 0.001*
4	93	285	3500	25 (27%)	16 (17%)	1.59	0.11

Table 6 reports the number and percentage of gamblers who withdrew money for each intensity group as well as the respective number for the matched observations for the ‘withdrawal’ message. For each intensity group, the percentage was larger among gamblers who read the ‘withdrawal’ message compared to the matched observations. Only in the second most gambling intense group was the difference not significant. The differences among the other three groups were significant.

**Table 6:** Percentage of gamblers withdrawing split into four groups based on the amount of money deposited seven days before. Results for the ‘withdrawal’ message

Intensity group	N	Minimum amount of money deposited seven days before	Maximum amount of money deposited seven days before	Number of observations with withdrawal	Number of matched observations with withdrawal	Z	*p*
1	311	8	125	52 (16%)	25 (8%)	3.3	0.001*
2	315	126	215	46 (15%)	40 (13%)	0.69	0.49
3	322	216	360	66 (20%)	34 (11%)	3.58	< 0.001*
4	323	364	4045	72 (22%)	32 (10%)	4.33	< 0.001*

## Discussion

Online gambling operators have access to gamblers and their data at any time. While less than 10% of gamblers ever seek treatment (Clarke et al., 2007; Suurvali et al., 2010), personalized messages can be delivered to every individual including those displaying risky or problematic gambling behavior. Past studies have shown that personalized messaging can lead to decreased spending on gambling (Auer & Griffiths, 2015, 2020). The present study tested whether nudging (Thaler & Sunstein, 2009) in the form of tailored personalized messages could be used to trigger a specific behavior (i.e., withdrawing money from their gambling account).

A sample of real-world Dutch online gamblers was utilized for the study. Given that gamblers were not randomly assigned to groups, a matched-pairs design was applied similar to previous studies using account-based tracking data (Auer & Griffiths, 2015, 2020, 2022b; Zhuang et al, 2018; Gainsbury et al., 2016). In total, 4049 gamblers read at least one message between October 1st 2022 and January 31st 2023. Because some gamblers read messages on one more than one day, the total number of observations was 4362. Out of these, 1663 were able to be assigned to a matched pair (38%). In previous studies, matched pairs could not be assigned to every player or observation (Auer & Griffiths, 2015, 2020, 2022b). In the present study, the matched (n = 1663) and unmatched (n = 2699) observations did not differ with respect to gambling intensity because the amount of money deposited was larger among the matched observations, but the amount of money bet was larger among the unmatched observations. Unmatched gamblers won more money than they wagered, and matched observations lost more money than they wagered. However, monetary loss is a volatile metric, especially as the median values for each gambler were based on a rather small number of records. Auer et al. (2012) described the behavior of the loss metric for a small number versus a large number of observations. For a large number of observations, the loss will converge towards the bet multiplied by the payout percentage. However, for a small number of observations the loss can vary substantially and does not reflect a player’s monetary gambling intensity.

In the present study, 8% of the gamblers for whom a match could be found were female and 16% of the gamblers for whom no match could be found were female. This could be due to a lower percentage of females among the pool of gamblers not reading personalized messages. The lower percentage could have led to less potential matches. Several previous studies have reported lower percentages of females being online gamblers compared to males (e.g., Columb & O’Gara, 2018; McCormack et al., 2014).

Gamblers received two types of personalized messages. One message (‘winning streak’) was triggered when gamblers won €250 the previous day. The second message (‘withdrawal’) was triggered when gamblers withdrew relatively little money compared to how much money they deposited during the previous 30 days. This was also reflected in the average amount of money won, amount of money deposited, and amount of money bet, in the two messages. Gamblers who read the ‘winning streak’ messages won more money than they deposited in the previous seven days. The amounts of money deposited and bet during the previous seven days was larger among gamblers reading the ‘withdrawal’ messages. Only gamblers who deposited at least €500 in a 30-day period received the ‘withdrawal’ messages.

The present study found that 38% of gamblers reading the ‘winning streak’ messages withdrew money from their gambling account on the same day. Only 14% among the matched pairs withdrew money. This supports the assumption that the message nudged gamblers to withdraw money from their gambling account. The study also found that 18% of gamblers reading the ‘withdrawal’ messages withdrew money from their gambling account on the same day. This percentage was lower compared to the ‘winning streak’ messages. One possible explanation is that gamblers reading the ‘withdrawal’ messages had won less money, and had actually lost money, compared to gamblers reading the ‘winning streak’ messages. This is also explained by the different criteria which trigger ‘winning streak’ and ‘withdrawal’ messages. Winning streak messages are sent to gamblers who won the day before and ‘withdrawal’ messages are sent to players who lost the day before. The less money gamblers have in their gambling account the less likely they can withdraw. The 18% of gamblers reading the ‘withdrawal’ messages was still significantly higher than the 10% withdrawing money among their matched pairs. Therefore, the ‘withdrawal’ messages appear to have had a significant impact on money withdrawal. Although previous studies have not measured money withdrawal, they have shown that personalized messages have a positive impact on reducing subsequent gambling behavior (Auer & Griffiths, 2015, 2020).

Gamblers who read personalized messages also withdrew larger amounts of money from their gambling accounts compared to gamblers who did not read personalized messages. This suggests that the personalized messages had an impact on both the likelihood to withdraw money as well as the amount of money which was withdrawn. This finding is further strengthened by the fact that the amount of money won or lost was used in the initial matching procedure.

The authors also wanted to investigate whether the level of monetary gambling intensity correlated with the effect of the personalized messages. Previous studies have found that gambling involvement is positively correlated with problem gambling (Binde et al., 2017; Gainsbury et al., 2015). Gamblers were categorized into four groups based on the amount of money deposited during the previous seven days for each message type. Among the 25% of gamblers with the highest amount of money deposited, there was no significant effect of the ‘winning streak’ message in the likelihood of withdrawing money from their gambling account. However, among the 25% of gamblers with the highest amount of money deposited, the percentages of gamblers withdrawing money was 27% among gamblers reading ‘winning streak’ messages and 17% for the matched observations. The withdrawal of money was therefore still quite different between the target group and control group, but was probably non-significant due to the small number of observations (i.e., the number of observations among the group of gamblers with the largest amount deposited was fairly low; n = 93). The ‘withdrawal’ message had a significant effect on the likelihood of withdrawing money among the group of gamblers who deposited the highest amount of money. Among this group of high intensity gamblers, 22% deposited who read a message compared to 10% among the respective matched observations.

The significant impacts of the personalized messages on the subsequent withdrawal of money are in line with the findings of previous research. Auer and Griffiths (2020) studied the impact of similar personalized messages on the amount bet and also found that gamblers who won and were nudged to withdraw money, bet less money on the day they read the message. The findings of the present study are also in line with Gainsbury et al.’s (2020) assumptions that behavioral economics research could be used to identify nudges that might help maintain healthy levels of gambling. The present study is the first to investigate the impact of personalized messages on the withdrawal of money and it showed that personalized messages can be a successful strategy to nudge players to withdraw money. Future studies should attempt to investigate nudging in other populations of gamblers and on different online gambling websites.

The present study has a number of limitations. First, the data were from only one online gambling operator from one specific country. Online gambling regulation and regulatory requirements vary greatly with respect to limit setting, self-exclusion, maximum spending limits, and types of games offered. Such differences might have a significant impact on the findings if the study was replicated in another region and/or with another gambling operator. Second, there was only a limited time period for which the data were available. Significant events which could have influenced the findings might have occurred during this time period. Third, the present study used a matched-pairs design. A majority of the target group players were discarded because they could not be matched with similar players that did not read messages. However, the gamblers who were unable to be matched might have reacted differently after reading the personalized messages compared to those who had suitable matches. Fourth, the present study did not investigate long-term effects of the personalized messages on gambling behavior or investigate the impact of the personalized messages on the amount of money bet or lost. Future studies should incorporate these outcome variables. Further research should also be carried out with gamblers from different operators and different countries. Future research should also apply a fully randomized experimental design in which players are randomly assigned to one of several conditions. Future research could also test the impact of normative feedback in the message texts as this might increase the likelihood of withdrawing money.

Gambling regulators in several countries (e.g., United Kingdom, Sweden, Spain, Netherlands, Germany) have now made it mandatory to provide feedback to players about gambling expenditure. This means that some gambling operators have to inform players about their losses after login or in dedicated sections of their online gambling website. The present study’s results could be used by policymakers and regulators to further extend responsible gaming requirements. Regulators could make it mandatory for personalized messages with specific nudges to be introduced as another responsible gambling tool to reduce gambling-related harm.

## Conclusion

The results of the present study suggest that personalized messages which nudge players to withdraw money from their online gambling account can be successful for some gamblers. Given that gamblers cannot gamble or overspend if the amount of money is limited, the present study’s findings suggest that these types of messages help in reducing gambling expenditure and may ultimately help in reducing gambling-related problems.

## Data Availability

The dataset used for this study was supplied by an online gambling operator and are commercially sensitive. Therefore, the dataset is not available to other researchers.

## References

[CR1] Arno, A., & Thomas, S. (2016). The efficacy of nudge theory strategies in influencing adult dietary behaviour: A systematic review and meta-analysis. *BMC Public Health,**16*(1), 676. 27475752 10.1186/s12889-016-3272-x PMC4967524

[CR2] Auer, M. M., & Griffiths, M. D. (2015). The use of personalized behavioral feedback for online gamblers: An empirical study. *Frontiers in Psychology,**6*, 1406. 26441779 10.3389/fpsyg.2015.01406 PMC4585278

[CR3] Auer, M., & Griffiths, M. D. (2017). Self-reported losses versus actual losses in online gambling: An empirical study. *Journal of Gambling Studies,**33*, 795–806. 27815667 10.1007/s10899-016-9648-0 PMC5579145

[CR4] Auer, M., & Griffiths, M. D. (2020). The use of personalized messages on wagering behavior of Swedish online gamblers: An empirical study. *Computers in Human Behavior,**110*, 106402. 10.1016/j.chb.2020.106402

[CR5] Auer, M., & Griffiths, M. D. (2022a). Attitude towards deposit limits and relationship with their account-based data among a sample of German online slots players. *Journal of Gambling Studies,**39*, 1319–1336. 36002706 10.1007/s10899-022-10155-1 PMC9401202

[CR6] Auer, M., & Griffiths, M. D. (2022b). The impact of personalized feedback interventions by a gambling operator on subsequent gambling expenditure in a sample of Dutch online gamblers. *Journal of Gambling Studies*. 10.1007/s10899-022-10162-2 36352314 10.1007/s10899-022-10162-2 PMC10175399

[CR7] Auer, M., & Griffiths, M. D. (2023). The relationship between structural characteristics and gambling behaviour: An online gambling player tracking study. *Journal of Gambling Studies,**39*, 265–279. 35553316 10.1007/s10899-022-10115-9 PMC9981537

[CR8] Auer, M., Schneeberger, A., & Griffiths, M. D. (2012). Theoretical loss and gambling intensity: A simulation study. *Gaming Law Review and Economics,**16*(5), 269–273. 10.1089/glre.2012.1655

[CR9] Barratt, M., Livingston, M., Matthews, S., & Clemens, S. (2014). Gaming machine density is correlated with rates of help-seeking for problem gambling: A local area analysis in Victoria, Australia. *Journal of Gambling Issues,**29*, 1–21. 10.4309/jgi.2014.29.16

[CR10] Benhsain, K., Taillefer, A., & Ladouceur, R. (2004). Awareness of independence of events and erroneous perceptions while gambling. *Addictive Behaviors,**29*, 399–404. 14732429 10.1016/j.addbeh.2003.08.011

[CR11] Binde, P., Romild, U., & Volberg, R. A. (2017). Forms of gambling, gambling involvement and problem gambling: Evidence from a Swedish population survey. *International Gambling Studies,**17*(3), 490–507. 10.1080/14459795.2017.1360928

[CR12] Braverman, J., Tom, M. A., & Shaffer, H. J. (2014). Accuracy of self-reported versus actual online gambling wins and losses. *Psychological Assessment,**26*(3), 865–877. 24708074 10.1037/a0036428

[CR13] Brooks, G., Ellis, T., & Lewis, C. (2008). Pachinko: A Japanese addiction? *International Gambling Studies,**8*, 193–205. 10.1080/14459790802168958

[CR14] Brosowski, T., Meyer, G., & Hayer, T. (2012). Analyses of multiple types of online gambling within one provider: An extended evaluation framework of actual online gambling behaviour. *International Gambling Studies,**12*(3), 405–419. 10.1080/14459795.2012.698295

[CR15] Chóliz, M. (2016). The challenge of online gambling: The effect of legalization on the increase in online gambling addiction. *Journal of Gambling Studies,**32*, 749–756. 26058374 10.1007/s10899-015-9558-6

[CR16] Chóliz, M., Marcos, M., & Lázaro-Mateo, J. (2019). The risk of online gambling: A study of gambling disorder prevalence rates in Spain. *International Journal of Mental Health and Addiction,**19*(2), 1–14.

[CR17] Chóliz, M., Marcos, M., & Lázaro-Mateo, J. (2021). The risk of online gambling: A study of gambling disorder prevalence rates in Spain. *International Journal of Mental Health and Addiction,**19*, 404–417. 10.1007/s11469-019-00067-4

[CR18] Clarke, D., Abbott, M., DeSouza, R., & Bellringer, M. (2007). An overview of help seeking by problem gamblers and their families including barriers to and relevance of services. *International Journal of Mental Health and Addiction,**5*, 292–306. 10.1007/s11469-007-9063-y

[CR19] Columb, D., & O’Gara, C. (2018). A national survey of online gambling behaviours. *Irish Journal of Psychological Medicine,**35*(4), 311–319. 30501670 10.1017/ipm.2017.64

[CR20] Cuzick, J. (1985). A Wilcoxon-type test for trend. *Statistics in Medicine,**4*(1), 87–90. 3992076 10.1002/sim.4780040112

[CR21] Dowling, N., Smith, D., & Thomas, T. (2005). Electronic gaming machines: Are they the ‘crack-cocaine’ of gambling? *Addiction,**100*(1), 33–45. 15598190 10.1111/j.1360-0443.2005.00962.x

[CR22] Effertz, T., Bischof, A., Rumpf, H.-J., Meyer, C., & John, U. (2018). The effect of online gambling on gambling problems and resulting economic health costs in Germany. *European Journal of Health Economics,**19*(7), 967–978. 10.1007/s10198-017-0945-z 29362900

[CR23] Emond, A. M., Griffiths, M. D., & Hollén, L. (2020). Problem gambling in early adulthood: A population-based study. *International Journal of Mental Health and Addiction,**20*, 754–770. 35368861 10.1007/s11469-020-00401-1 PMC8930883

[CR24] Focal Research (2004). *2003 NS VL responsible gaming features evaluation*. *Final Report.* Focal Research Consultants Ltd.

[CR25] Gainsbury, S. M., Black, N., Blaszczynski, A., Callaghan, S., Clancey, G., Starcevic, V., & Tymula, A. (2020). Reducing internet gambling harms using behavioral science: A stakeholder framework. *Frontiers in Psychiatry,**11*, 598589. 33381059 10.3389/fpsyt.2020.598589 PMC7768631

[CR72] Gainsbury, S. M., Liu, Y., Russell, A. M., & Teichert, T. (2016). Is all Internet gambling equally problematic? Considering the relationship between mode of access and gambling problems. *Computers in Human Behavior*, *55*, 717–728.

[CR27] Gainsbury, S. M., Russell, A., Wood, R., Hing, N., & Blaszczynski, A. (2015). How risky is internet gambling? A comparison of subgroups of Internet gamblers based on problem gambling status. *New Media & Society,**17*(6), 861–879. 10.1177/1461444813518185

[CR28] Gillingham, K., & Tsvetanov, T. (2018). Nudging energy efficiency audits: Evidence from a field experiment. *Journal of Environmental Economics and Management,**90*, 303–316. 10.1016/j.jeem.2018.06.009

[CR29] González-Roz, A., Fernández-Hermida, J. R., Weidberg, S., Martínez-Loredo, V., & Secades-Villa, R. (2017). Prevalence of problem gambling among adolescents: A comparison across modes of access, gambling activities, and levels of severity. *Journal of Gambling Studies,**33*, 371–382. 27785589 10.1007/s10899-016-9652-4

[CR30] Griffiths, M. D. (2008). *Impact of high-stake, high-prize gaming machines on problem gambling: overview of research findings*. Report prepared for the Great Britain Gambling Commission.

[CR31] Griffiths, M. D. (1993). Fruit machine gambling: The importance of structural characteristics. *Journal of Gambling Studies,**9*, 101–120. 10.1007/BF01014863

[CR32] Griffiths, M. D. (2003). Internet gambling: Issues, concerns, and recommendations. *CyberPsychology & Behavior,**6*(6), 557–568. 14756922 10.1089/109493103322725333

[CR33] Griffiths, M. D., & Calado, F. (2022). Gambling disorder. In H. Pontes (Ed.), *Behavioral addictions: Conceptual, clinical, assessment, and treatment approaches* (pp. 1–29). Springer.

[CR35] Griffiths, M., Parke, A., Wood, R., & Parke, J. (2006). An overview of psychosocial impacts. *UNLV Gaming Research & Review Journal,**10*, 27–39.

[CR36] Griffiths, M. D., Parke, J., Wood, R. T. A., & Rigbye, J. (2010). Online poker gambling in university students: Further findings from an online survey. *International Journal of Mental Health and Addiction,**8*, 82–89. 10.1007/s11469-009-9203-7

[CR37] Griffiths, M. D., Wardle, H., Orford, J., Sproston, K., & Erens, B. (2009). Sociodemographic correlates of internet gambling: Findings from the 2007 British gambling prevalence survey. *CyberPsychology and Behavior,**12*(2), 199–202. 19072080 10.1089/cpb.2008.0196

[CR38] Håkansson, A., & Åkesson, G. (2022). Multi-operator self-exclusion as a harm reduction measure in problem gambling: Retrospective clinical study on gambling relapse despite self-exclusion. *JMIR Mental Health,**9*(8), e37837. 35984678 10.2196/37837 PMC9440409

[CR39] Heirene, R. M., & Gainsbury, S. M. (2021). Encouraging and evaluating limit-setting among on-line gamblers: A naturalistic randomized controlled trial. *Addiction,**116*(10), 2801–2813. 33751702 10.1111/add.15471

[CR40] Heirene, R. M., Wang, A., & Gainsbury, S. M. (2022). Accuracy of self-reported gambling frequency and outcomes: Comparisons with account data. *Psychology of Addictive Behaviors,**36*(4), 333–346. 34914407 10.1037/adb0000792

[CR41] Ivanova, E., Magnusson, K., & Carlbring, P. (2019). Deposit limit prompt in online gambling for reducing gambling intensity: A randomized controlled trial. *Frontiers in Psychology,**10*, 639. 31001160 10.3389/fpsyg.2019.00639 PMC6455077

[CR42] Ladouceur, R., & Sevigny, S. (2003). Interactive messages on video lottery terminals and persistence in gambling. *Gambling Research,**15*, 45–50.

[CR43] Lawley, D. N. (1938). A generalization of Fisher’s z test. *Biometrika,**30*(1/2), 180–187. 10.2307/2332232

[CR70] Lawn, S., Oster, C., Riley, B., Smith, D., Baigent, M., & Rahamathulla, M. (2020). A literature review and gap analysis of emerging technologies and new trends in gambling. *International Journal of Environmental Research and Public Health*, *17*(3), 744. 10.3390/ijerph17030744 PMC7036923 31979364

[CR71] Livingstone, C., & Woolley, R. (2008). The relevance and role of gaming machine games and game features on the play of problem gamblers. A*ustralian Institute for Primary Care and Ageing.*

[CR44] MacFarland, T. W., & Yates, J. M. (2016). Mann–Whitney U Test. *Introduction to Nonparametric Statistics for the Biological Sciences Using R*, (pp. 103–132). Springer.

[CR45] McBride, J., & Derevensky, J. (2009). Internet gambling behavior in a sample of online gamblers. *International Journal of Mental Health and Addiction,**7*, 149–167. 10.1007/s11469-008-9169-x

[CR46] McCormack, A., Shorter, G. W., & Griffiths, M. D. (2014). An empirical study of gender differences in online gambling. *Journal of Gambling Studies,**30*, 71–88. 23097131 10.1007/s10899-012-9341-x

[CR47] Monaghan, S. (2008). Review of pop-up messages on electronic gaming machines as a proposed responsible gambling strategy. *International Journal of Mental Health and Addiction,**6*, 214–222. 10.1007/s11469-007-9133-1

[CR48] Mora-Salgueiro, J., García-Estela, A., Hogg, B., Angarita-Osorio, N., Amann, B. L., Carlbring, P., & Colom, F. (2021). The prevalence and clinical and sociodemographic factors of problem online gambling: A systematic review. *Journal of Gambling Studies,**37*, 899–926. 33512624 10.1007/s10899-021-09999-w

[CR49] Newall, P. W. (2019). Dark nudges in gambling. *Addiction Research & Theory,**27*(2), 65–67. 10.1080/16066359.2018.1474206

[CR50] Petry, N. M. (2006). Internet gambling: An emerging concern in family practice medicine? *Family Practice,**23*, 421–426. 16621919 10.1093/fampra/cml005

[CR51] Schellink, T. & Schrans, T. (2002) *Atlantic lottery corporation video lottery responsible gaming feature research: Final report.* Halifax, Nova Scotia. Focal Research Consultants.

[CR52] St-Pierre, R. A., Walker, D. M., Derevensky, J., & Gupta, R. (2014). How availability and accessibility of gambling venues influence problem gambling: A review of the literature. *Gaming Law Review and Economics,**18*(2), 150–172. 10.1089/glre.2014.1824

[CR53] Suurvali, H., Hodgins, D. C., & Cunningham, J. A. (2010). Motivators for resolving or seeking help for gambling problems: A review of the empirical literature. *Journal of Gambling Studies,**26*, 1–33. 19768660 10.1007/s10899-009-9151-y

[CR54] Thaler, R. H., & Sunstein, C. R. (2009). *Nudge: Improving conditions about health, wealth and happiness*. Penguin.

[CR55] Volberg, R. A., McNamara, L. M., & Carris, K. L. (2018). Risk factors for problem gambling in California: Demographics, comorbidities and gambling participation. *Journal of Gambling Studies,**34*(2), 361–377. 28685275 10.1007/s10899-017-9703-5

[CR56] Wardle, H., Moody, A., Griffiths, M. D., Orford, J., & Volberg, R. (2011). Defining the online gambler and patterns of behaviour integration: Evidence from the British gambling prevalence survey 2010. *International Gambling Studies,**11*, 339–356. 10.1080/14459795.2011.628684

[CR57] Welte, J. W., Wieczorek, W. F., Barnes, G. M., Tidwell, M. C., & Hoffman, J. H. (2004). The relationship of ecological and geographic factors to gambling behavior and pathology. *Journal of Gambling Studies,**20*, 405–423. 15577275 10.1007/s10899-004-4582-y

[CR58] Wijesingha, R., Leatherdale, S. T., Turner, N. E., & Elton-Marshall, T. (2017). Factors associated with adolescent online and land-based gambling in Canada. *Addiction Research and Theory,**25*(6), 525–532. 10.1080/16066359.2017.1311874

[CR59] Wohl, M. J., Davis, C. G., & Hollingshead, S. J. (2017). How much have you won or lost? Personalized behavioral feedback about gambling expenditures regulates play. *Computers in Human Behavior,**70*, 437–445. 10.1016/j.chb.2017.01.025

[CR60] Yazdi, K., & Katzian, C. (2017). Addictive potential of online-gambling. A prevalence study from Austria. *Psychiatria Danubina,**29*(3), 376–378. 28949319 10.24869/psyd.2017.276

[CR61] Zhuang, X. Y., Wong, D. F. K., Ng, T. K., Jackson, A. C., Dowling, N. A., & Lo, H. H. M. (2018). Evaluating the effectiveness of an integrated cognitive-behavioural intervention (CBI) model for male problem gamblers in Hong Kong: A matched-pair comparison design. *Journal of Gambling Studies,**34*, 969–985. 29357020 10.1007/s10899-018-9747-1

